# Instance segmentation convolutional neural network based on multi-scale attention mechanism

**DOI:** 10.1371/journal.pone.0263134

**Published:** 2022-01-27

**Authors:** Wang Gaihua, Lin Jinheng, Cheng Lei, Dai Yingying, Zhang Tianlun

**Affiliations:** 1 School of Electrical and Electronic Engineering, Hubei University of Technology, Wuhan, China; 2 Hubei Key Laboratory for High-Efficiency Utilization of Solar Energy and Operation Control of Energy Storage System, Hubei University of Technology, Wuhan, China; Hefei University of Technology, CHINA

## Abstract

Instance segmentation is more challenging and difficult than object detection and semantic segmentation. It paves the way for the realization of a complete scene understanding, and has been widely used in robotics, automatic driving, medical care, and other aspects. However, there are some problems in instance segmentation methods, such as the low detection efficiency for low-resolution objects and the slow detection speed of images with complex backgrounds. To solve these problems, this paper proposes an instance segmentation method with multi-scale attention, which is called a Hybrid Kernel Mask R-CNN. Firstly, the hybrid convolution kernel is constructed by combining different kernels and groups, which can complement each other to extract rich information. Secondly, a multi-scale attention mechanism is designed by assign weights to different convolution kernels, which can retain more important information. After the introduction of our strategy, the network is more inclined to focus on the low-resolution objects in the image. The proposed method achieves the best accuracy over the anchor-based method. To verify the universality of the model, we test Hybrid Kernel Mask R-CNN on Balloon, xBD and COCO datasets. The test results exceed the state of art methods. And the visualization results show our method can extract low-resolution objects effectively.

## 1 Introduction

Instance segmentation is a complex issue and one of the most challenging computer vision tasks, which can perform instance segmentation by detecting objects and predicting pixel-level instances on objects.

Instance segmentation can be roughly divided into segmentation-based methods and detection-based methods. Segmentation-based methods predict pixel-level categories and then aggregate the same class to achieve the final instance segmentation results. Bai et al. [1] predict pixel-level energy values and group by using watershed algorithms. Kirillov et al. [2] add boundary detection information during the clustering procedure to improve the accuracy of models. Detection-based methods can trace back to DeepMask [3], which generates instance masks by using sliding windows and predicts the mask by utilizing some detectors, such as R-FCN [4]. DeepMask cannot achieve superior performance in instance segmentation due to redundant feature representation and lack of local-cohere. Dai et al. [5] propose instance-sensitive FCNs to generate the position-sensitive maps and assemble them to obtain the final masks. Mask R-CNN [6], a simple and effective method for instance segmentation, generates a binary mask for each class independently. Based on Faster R-CNN [7] a fully convolutional network (FCN) is used to achieve semantic segmentation by adding mask branching. To fuse different level features, a feature pyramid network (FPN) [8] is used to capture more details from different levels of feature maps. Liu et al. [9] propose the Path Aggregation Network (PANET), which adds a bottom-up path on the basis of FPN to promote information flow. Li et al. [10] propose TridentNet to generate scale-specific feature maps with a uniform representational power. These methods usually use fixed kernel size, 3×3. When kernel size increases, the amount of computation increases rapidly. Do larger kernels always improve the efficiency of CNNs?

Some studies [11–14] show that using larger kernels, such as 5x5, 7x7 and 9x9, can achieve higher accuracy. If kernel size continues to increase or is equal to the input resolution, which is the same as a fully-connected network, the network has more parameters and complexity and the performance of the network is inferior [15]. Therefore, networks with a single kernel and oversized kernel are not an optimal solution.

Inspired by the above principles and observations, this paper proposes Hybrid Kernel Mask R-CNN (HKMask). The main contributions are as follows: (1) In order to capture the scales variability, a hybrid kernel module is constructed, which contains different levels of kernels with varying size and depth. And for the corresponding kernels, it uses different groups. (2) Based on the Squeeze-and-Excitation Networks, the model provides an improved channel attention module that preserves more important information through the idea of shortcut connection.

## 2 Related work

### 2.1 Instance segmentation based on mask R-CNN

Mask R-CNN [6], one of the detection-based methods, which changes ROI pooling to a quantization-free layer called ROIAlign and generates a binary mask for each class independently. It has achieved the best result of a single model in the 2018 COCO [16] Instance Segmentation Challenge. Many methods were designed to improve the Mask R-CNN [6]. Liu et al. [9] presented adaptive feature pooling to boost information flow in a proposal-based instance segmentation framework. Huang et al. [17] proposed Mask Scoring R-CNN to improve the predictive quality of instance masks. Cai et al. [18] proposed a classic and powerful architecture called Cascade R-CNN, which uses progressive refinement of predictions and adaptive handling of training distributions to boost performance. Lee et al. [19] proposed CenterMask, which uses an anchor-free instance segmentation to alleviate the saturation problem. In this paper, the method is also based on Mask RCNN. It adds a hybrid kernel module and multi-scale attention to Mask RCNN.

### 2.2 Channel attention module

The attention mechanism is to select the information that is more critical to the current task from a large amount of information. Relevant studies have shown that the integration of attention mechanism and CNNs helps to enhance the spatial correlation of feature maps [20–22]. Bell et al. [23] introduced a spatial attention mechanism into the architecture to improve the spatial relevance of the model. SENet [24] is a representative channel attention method in CNNS. And it uses the global average pooling to get the feature information between each channel and realizes the spatial information compression. Then two fully connected layers are used to obtain more nonlinear connections between different channels. These can retain useful information and suppress irrelevant information.

## 3 Model

**Fig 1:**
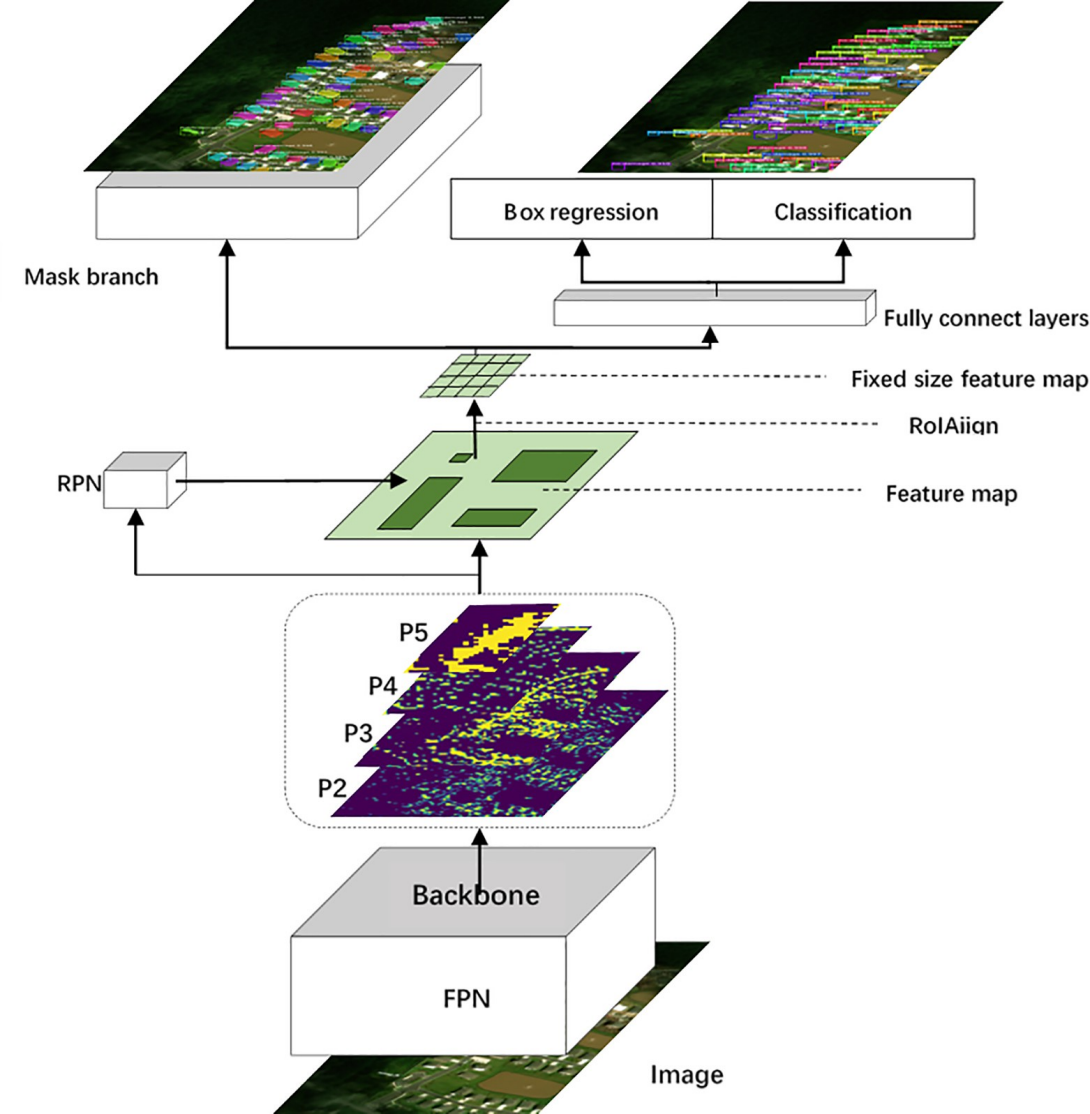
The HKMask framework for instance segmentation.

Multi-scale convolution kernel and attention mechanism are introduced into the feature extraction network for feature extraction (Fig 1). And then the extracted features are sent into the RPN network to generate proposal. Finally, ROIAlign unifies the scale and completes the instance segmentation through the semantic segmentation branch and target detection branch.

### 3.1 Hybrid kernel

**Fig 2:**
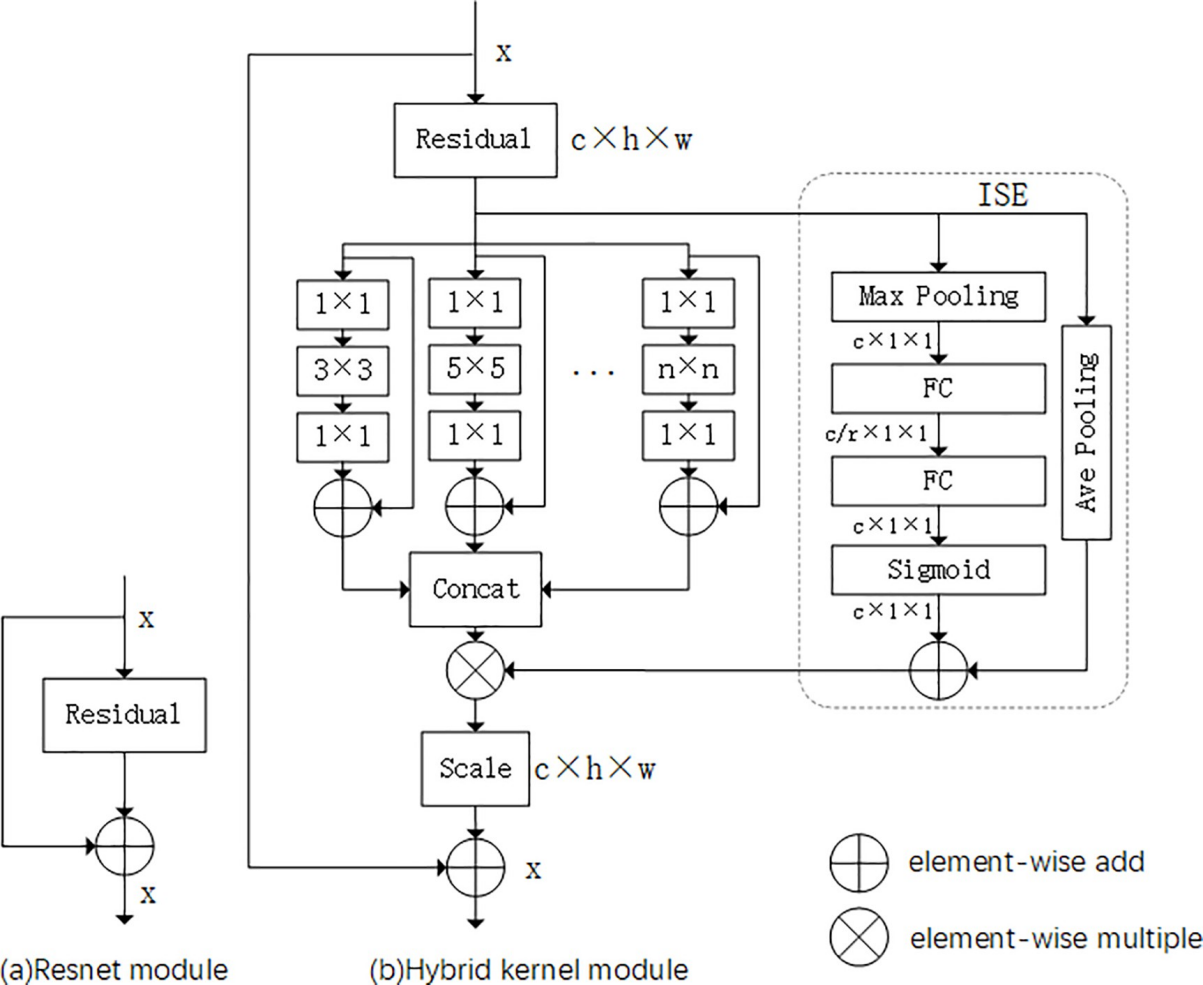
The schema of the original residual module (a) and the hybrid kernel module (b). Hybrid kernel module introduces attention mechanism and mixed convolution on the basis of the original Resnet module.

ResNet-50 module (Fig 2(A)) contains only one single convolution kernel, which is 3×3, and the kernel depth gradually increases with the deepening of network. In order to reduce the complexity and computation, we introduce a hybrid kernel module (Fig 2(B)) based on ResNet-50. In this module, kernels of different sizes and depths are used. The hybrid kernel module can be regarded as an alternative to the ordinary convolution.

#### 3.1.1 Kernel size

In the feature extraction process, the use of large kernel will skip a lot of local information, which is not conducive to the process of feature extraction. We utilize different branches to construct hybrid multi-scale module, including 3×3, 5x5, 7x7 and 9x9. Not only can the receptive field of convolution kernel be expanded, but also the information extracted by different kernels can complement each other.

#### 3.1.2 Kernel group

Hybrid kernel module divides channels into groups. It uses different sized kernels for each group in feature extraction, while parallel computation can be done independently between groups. Let *T*^(*h*,*w*,*c*)^ denotes the input tensor, where H is the space height, W is the space width and C is the channel size. Let *W*^(*k*,*k*,*c*,*m*)^ denotes a standard convolutional kernel, where k×k is the kernel size, c is the input channel size and m is the channel multiplier. we divide the input tensor *T*^(*h*,*w*,*c*)^ and convolution kernel *W*^(*k*,*k*,*c*,*m*)^ into G group. They are $\left[t^{\left(h,w,c_{1}\right)},t^{\left(h,w,c_{2}\right)},...,t^{\left(h,w,c_{G}\right)}\right]$ and $\left[w^{\left(k_{1},k_{1},c_{1},m\right)},w^{\left(k_{1},k_{1},c_{2},m\right)},...,w^{\left(k_{1},k_{1},c_{G},m\right)}\right]$, c_1_+c_2_+…+c_*G*_ = *c*.The k is set to value, ranges from [1, 4]. The output of each group is expressed by Eq 1.


$$Y_{x,y,z_{n}}^{n}=\underset{-\frac{kn}{2}\leq i\leq \frac{kn}{2},-\frac{kn}{2}\leq j\leq \frac{kn}{2}}{\sum }t_{x+i,y+i,\frac{z}{m}}^{\left(h,w,c_{n}\right)}\cdot W_{i,j,z}^{\left(k_{n},k_{n},c_{n}\right)},\;z=1,2,\ldots ,m\cdot c_{n}$$ (1)

The final output is shown in Eq 2.


$$Y_{x,y,z}^{n}=Concat(Y_{x,y,z_{1}}^{1},Y_{x,y,z_{2}}^{2},...,Y_{x,y,z_{G}}^{G})$$ (2)

z = *z*_1_+*z*_2_+…+*z*_*G*_ = *m*•*c*. If the number of groups increases, the number of parameters and the computational cost will decrease. It can alleviate the computational cost of large kernel.

#### 3.1.3 Kernel number

In order to facilitate the embedding of hybrid kernel block into other deep CNNs, we adopt the same configuration information in ResNet and just replace the target ResNet module with ours. After the replacement, the generated network is named hybrid kernel module-50. Different numbers of convolutional kernels are used in FPN. The number of convolution kernels decreases gradually with the network deepens. The detailed kernel groups are shown in Table 1.

**Table 1:** Details of ResNet-50 and hybrid kernel module-50.

Stage	Input	ResNet-50	Hybrid kernel module-50	Output
1	224×224	7×7, 64, s = 2	7×7, 64, s = 2	112×112
	112×112	3×3 *max pool*, *s* = 2	3×3 *max pool*, *s* = 2	56×56
2	56×56	$[\begin{array}{c} 1\times 1,64\\ 3\times 3,64\\ 1\times 1,256 \end{array}]\times 3,s=1$	$[\begin{array}{c} 1\times 1,64\\ \left[\begin{array}{c} 9\times 9,16,G=1\\ 7\times 7,16,G=1\\ 5\times 5,16,G=2\\ 3\times 3,16,G=4 \end{array}\right]\\ 1\times 1,256 \end{array}]\times 3,s=1$	56×56
3	56×56	$[\begin{array}{c} 1\times 1,128\\ 3\times 3,128\\ 1\times 1,512 \end{array}]\times 4,s=2$	$[\begin{array}{c} 1\times 1,128\\ \left[\begin{array}{c} 7\times 7,64,G=2\\ 5\times 5,32,G=2\\ 3\times 3,32,G=4 \end{array}\right]\\ 1\times 1.512 \end{array}]\times 4,s=2$	28×28
4	28×28	$[\begin{array}{c} 1\times 1,256\\ 3\times 3,256\\ 1\times 1,1024 \end{array}]\times 6,s=2$	$[\begin{array}{c} 1\times 1,256\\ \left[\begin{array}{c} 5\times 5,128,G=2\\ 3\times 3,128,G=2 \end{array}\right]\\ 1\times 1,1024 \end{array}]\times 6,s=2$	14×14
5	14×14	$[\begin{array}{c} 1\times 1,512\\ 3\times 3,512\\ 1\times 1,2048 \end{array}]\times 3,s=2$	$[\begin{array}{c} 1\times 1,512\\ \left[3\times 3,512,G=1\right]\\ 1\times 1,2048 \end{array}]\times 3,s=2$	7×7

### 3.2 Attentional mechanism

**Fig 3:**
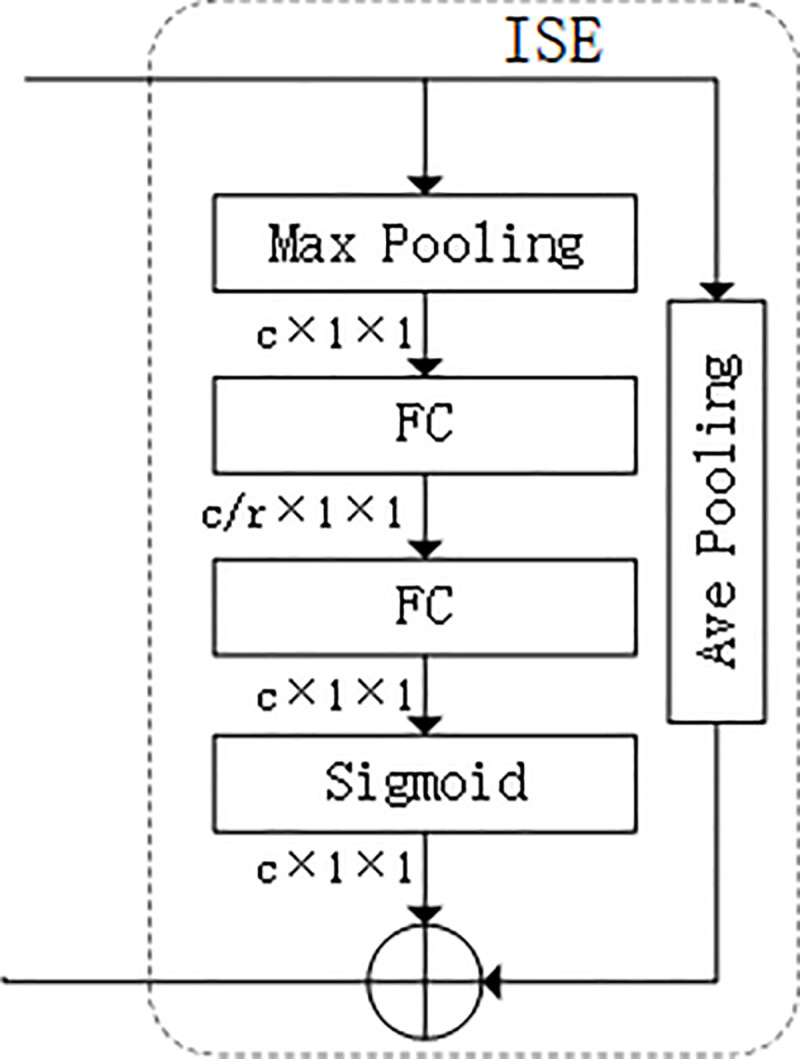
The improved squeeze-excitation networks. The max pooling is used to retain texture information and the average pooling retains global information of the feature map.

The Improved Squeeze-Excitation Networks (ISE) (Fig 3) is shown. The construction of this module is mainly inspired by the shortcut connection in ResNet. Max pooling encodes the most significant part of the image and average pooling encodes global statistics. We replace the average pooling of SENet with the max pooling, which is used to retain texture information in the feature map. To reduce the parameter error of the convolution and preserve more background information, the output is added with the average pooling through a shortcut connection.

The advantage of this strategy is that it not only preserves the ability of the original network to obtain the nonlinear relationship of the channel but also preserves more useful information about objects. In order to facilitate the embedding of ISE block into other deep CNNs, we adopt the same configuration information in SENet and just replace the target module SE block with ours.

## 4 Experiment

To prove the generalization of our model, we will evaluate the validity of HKMask with the methods [12, 13, 25] on Balloon, xBD and COCO datasets. The hardware configuration of this experiment is as follows: Intel(R) Core (TM) i9-9900K CPU, 64.0GB running memory, NVIDIA Quadro P2200 graphics card. Under Windows system, TensorFlow is used as the development platform. All pre-trained models we used are publicly available.

### 4.1 Datasets

To verify the universality of the network, we test the proposed method on three public datasets respectively. The selected datasets are Balloon, xBD and COCO.

**Fig 4:**
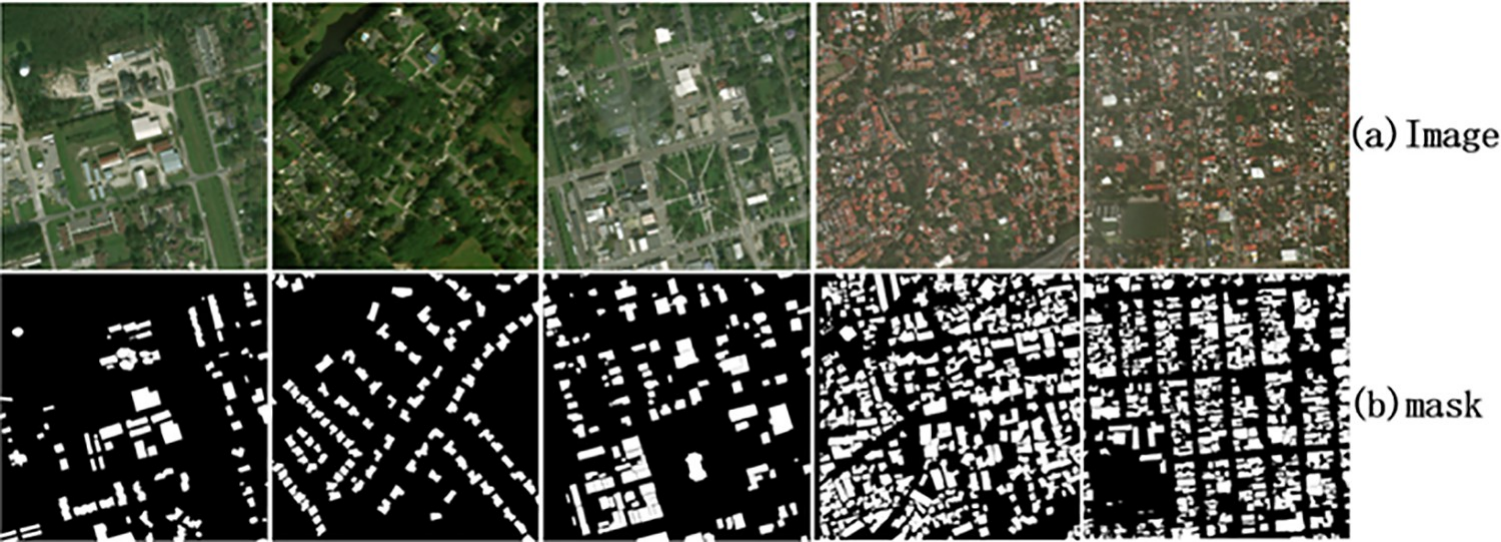
Original remote sensing image (a) and corresponding binary image (b).

(1) The Balloon dataset is the public dataset for target detection. This dataset contains 76 images, all of which are 1024*768 in size. The polygon position information of all Balloons is marked in the tag file, which can be used for target location and semantic segmentation.
(2) XBD has more than 850,000 architectural polygons from six different natural disasters around the world. XBD dataset contains 22068 images, all of which are high-resolution satellite remote sensing images of 1024*1024. Nineteen different events were marked, including earthquakes, floods and wildfires, etc. According to the label provided in xBD dataset, we generate a binary image (Fig 4) corresponding to each image. The binary image is used as a benchmark.
(3) COCO is a large and rich dataset for object detection, segmentation and caption. COCO contains 123K images with 80-class instance labels. Targeted at scene understanding, this dataset is mainly taken from complex scenes, Images include 91 categories of targets, 328,000 images and 2,500,000 labels. There are more than 330,000 images, 200,000 of which are labeled and the total number of individuals is more than 1.5 million. All models are trained on COCO 2017train (115K images) and the ablation study is carried out on COCO 2017val (5K images).

### 4.2 Experimental settings

We adopt ResNet-50 as the initial model. To make a fair comparison, we set the same hyper-parameters in Mask R-CNN. Specifically, we use randomly sampled images with short edges from {800,1024} for training and the number of images processed on each GPU is the default 2. The model is trained and optimized using the SGD. The learning rate is 0.01 and the step size is set to 100 during each iteration.

We use the model inference time of one thread (one batch size) as the training time. Test time is the average inference time of each image. The calculation process of precision is shown in Eq 3.


$$\mathrm{Precision}=\frac{T\mathrm{rue}\;\mathrm{positives}}{T\mathrm{rue}\;\mathrm{positives}+F\mathrm{alse}\;\mathrm{positives}}$$ (3)

We use COCO box average precision (AP), AP at IoU 0.5 (AP_50_) and 0.75 (AP_75_) as evaluation metrics.

### 4.3 Results and ablation study

**Fig 5:**
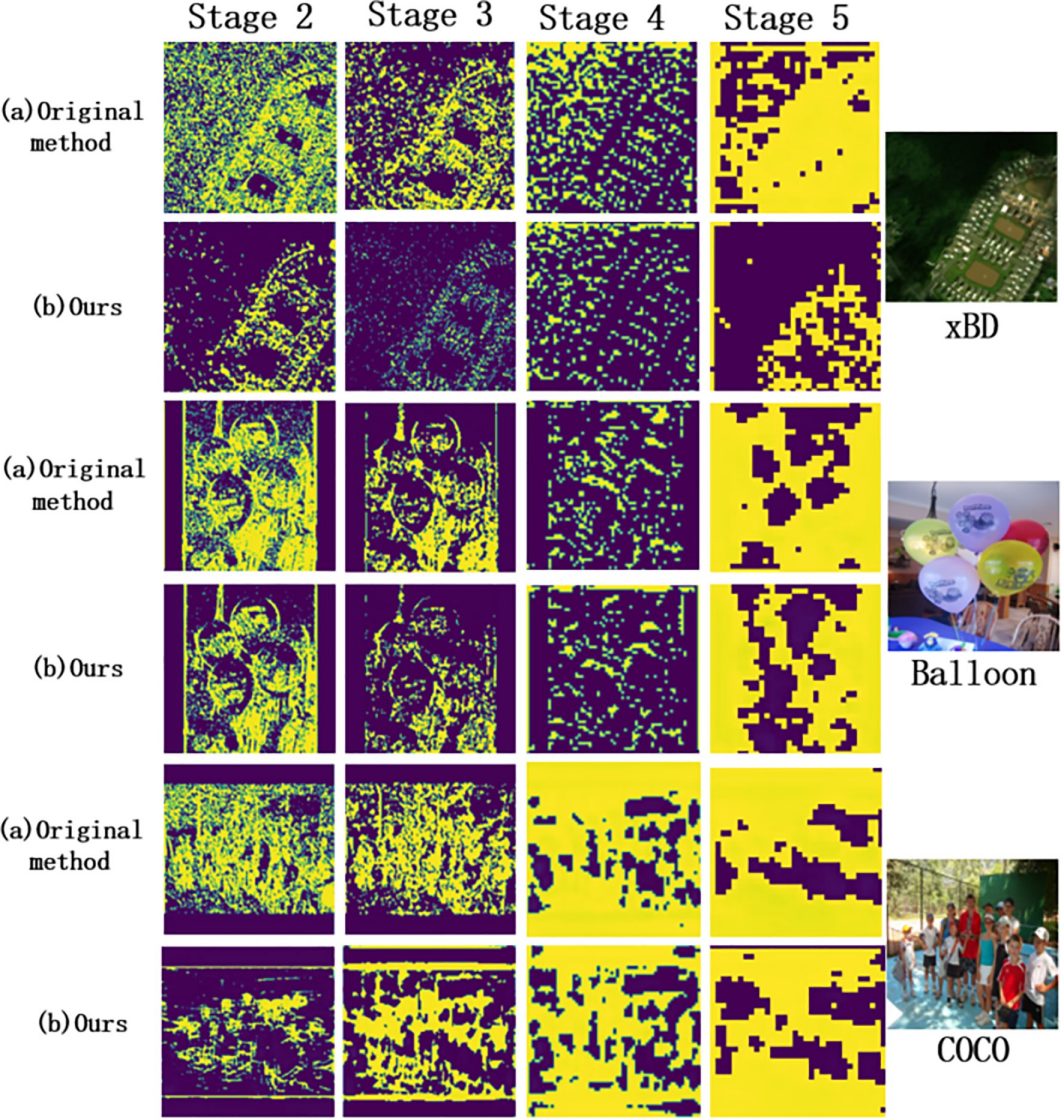
Feature maps of three datasets in different stages. Compared to the original method, ours has a significant suppressing effect on unrelated background pixels, while enhancing the pixels of the target instance. This is conducive to the convergence speed in the process of model training.

The feature maps of the original method and ours are shown respectively (Fig 5). The Stage in the figure represents the stage number. The higher number of stages is, the more details the information is focused on. According to the comparison of feature maps at each stage, it can be seen that the feature information extracted from the original method is greatly influenced by the background. The feature map appears a lot of irrelevant interference features, which directly leads to a lot of redundant calculations in the following process. HKMask introduces ISE to enhance the useful channels and suppress the irrelevant channels. By enhancing the feature learning process, the size and computation of the model can be reduced and the model training is accelerated.

Table 2 shows the AP gains of ISE and the comparison of processing speed on different datasets. As shown in Table 2, ISE can effectively shorten the image testing time on different datasets by 3.6%, 1.4% and 4.2%, respectively. After the introduction of ISE, the accuracy of the model is better than the baseline, reaching 82.60%, 60.5% and 58.57% AP_50_ on the three datasets, respectively. This not only means that ISE can shorten the testing time of the model, but also verifies that this module can improve the efficiency of feature extraction.

**Table 2:** The gains of ISE component in our design.

Dataset	ISE	AP	AP_50_	Train time(s)	Test speed(s)
Balloon	×	**72.75**	82.36	**145.85**	0.674
	**√**	72.41	**82.60**	157.01	**0.650**
COCO	×	34.7	54.4	**140.89**	1.348
	**√**	**37.4**	**60.5**	144.98	**1.329**
xBD	×	23.31	55.65	291.24	0.929
	**√**	**26.34**	**58.57**	**260.38**	**0.890**

Based on the framework of ResNet-50-FPN, results are reported on Balloon, COCO 2017val and xBD respectively.

Table 3 shows the AP gains of hybrid kernel and the comparison of parameters and complexity on different datasets. It is not difficult to find that the hybrid kernel has lower complexity and less computation, which is beneficial to the efficiency of the model. On COCO and xBD datasets, the hybrid kernel improves the baseline by 6% and 2.2% AP_50_ respectively. But on Balloon dataset, the AP50 dropped by 0. 3% AP. This happens because our strategy focuses on dense and objects with small resolution. If there are some instance objects of different scales in the image, the hybrid kernel shows better feature extraction. For images with a single instance, the module has a bad effect on accuracy.

**Table 3:** The gains of hybrid kernel component in our design.

Dataset	Hybrid Kernel	AP	AP_50_	Params	GFLOPs
Balloon	×	**72.8**	**82.4**	25.56	3.53
	**√**	70.9	82.1	24.85	3.27
COCO	×	34.7	54.4	25.56	3.53
	**√**	**37.0**	**60.4**	24.85	3.27
xBD	×	23.3	55.7	25.56	3.53
	**√**	**25.5**	**57.9**	24.85	3.27

Based on the framework of ResNet-50-FPN, results are reported on Balloon, COCO 2017val and xBD respectively.

We also compare HKMask with the recently proposed instance segmentation methods. As shown in Table 4, we comprehensively evaluate our method on COCO 2017val. From Table 4, it can be seen that the original Mask R-CNN is only 34.7% in box AP. After replacing the backbone with DarkNet-53, the model achieved an accuracy of 36.2% AP, 1.5% AP higher than the baseline. Although DarkNet-53 has a 1.5% AP improvement over ResNet-50, the image processing speed and memory utilization are much lower than ResNet-50 [32]. Due to the experimental environment, equipment conditions and other reasons, most of the models are tested on ResNet-50. In this case, the optimal performance of each model in the corresponding paper has not been restored. Therefore, it conducts a relatively optimized comparative experiment: that is, the above algorithms are reproduced under the same equipment and the same hyper-parameters. And the proposed algorithm achieves the best performance.

**Table 4:** Quantitative results on COCO 2017val.

Method	backbone	Epochs	AP	AP_50_	AP_75_
FCIS[26]	ResNet-101	12	29.5	51.5	30.2
Mask R-CNN	ResNet-50	36	34.7	54.4	37.7
Mask R-CNN	DarkNet-53[27]	36	36.2	57.8	39.4
YOLACT-550[28]	ResNet-50	48	29.8	49.4	31.5
YOLACT-550	ResNet-101	48	32.0	52.1	34.2
TensorMask[29]	ResNet-50	72	35.5	57.3	37.4
ConInst[30]	ResNet-50	36	39.7	58.8	43.1
BlendMask[31]	ResNet-50	36	**40.3**	59.2	**43.7**
HKMask(ours)	ResNet-50	36	37.8	**61.8**	41.8

ConInst and BlendMask are implemented with Detectron2 and the object detection box AP (%) are reported.

As can be seen from Tables 2 and 3, on COCO dataset, ISE and hybrid kernel have improved 2.7% and 2.3% AP respectively. However, after the aggregation of the two modules, the total output performance of the network is up to 37.8% AP, which is better than any single module. On the premise of introducing as few parameters as possible, our strategy aims at feature enhancement and background suppression, which is conducive to the efficiency of the anchor generation. The proposed method achieves the best accuracy over the anchor-based method. However, BlendMask, the mainstream instance segmentation method without anchor, is still in a leading position in AP. After the introduction of our strategy, the network is more inclined to focus on the low-resolution objects in the image. Compared with the recent instance segmentation methods, HKMask achieves the highest AP_50_ without longer training schedules needed. Specifically, HKMask achieves 61.8% AP_50_ after 36 training epochs, while all three metrics exceeded the baseline model Mask R-CNN. Although HKMask has some advantages in small target detection, it is still an anchor-based method in essence, and its simplicity and efficiency are still slightly lower than the current instance segmentation method based on FCOS.

**Fig 6:**
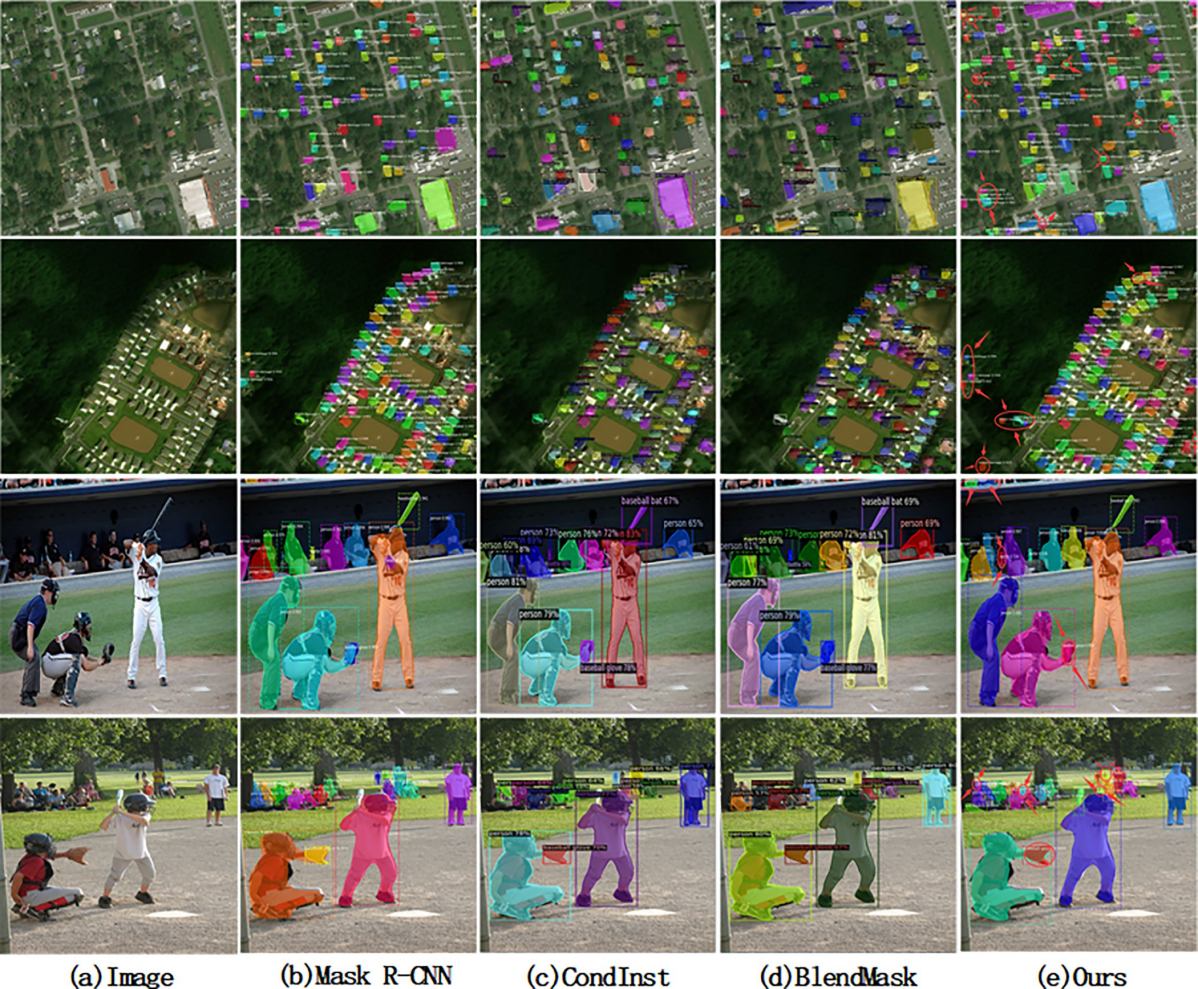
Qualitative result of different methods on xBD and COCO datasets.

To better understand HKMask, we provide some ablation experiments and the visualization results (Fig 6).

The qualitative results (Fig 6) of HKMask on COCO val2017 are shown. We mark the significant change with red circles. For low-resolution instances, other methods have cases of missing detection. In remote sensing images, it is not difficult to find that the background occupies most pixels, and advanced detectors, such as BlendMask, also have the situation of missing detection. Multi-scale convolution is beneficial to feature extraction at different scales, and the introduction of attention mechanism enhances the feature expression of detected objects. Therefore, our method has a better ability to extract low-resolution instance objects than other methods and solve the problem of missing detection, especially in the dataset with complex backgrounds.

## 5 Conclusion

In this paper, the current popular instance segmentation methods and attention mechanisms are discussed. Based on summarizing previous studies, an innovative model combining multi-scale convolution and attention mechanism is proposed to improve the model’s ability to detect instance objects with small resolution. The core module of HKMask is named hybrid kernel module. Specifically, the core of the proposed module is mainly as follows:

1. Multi-scale Convolution: The multi-scale convolution module is used to capture detection targets at different scales.
  Since different layers of FPN contain different semantic information, HKMask contains different levels of kernels with varying sizes and depths. In this way, the model can efficiently extract objects of different scales from images without introducing extra computation.
2. Attentional Mechanism: On the basis of Squeeze-and-Excitation Networks, HKMask introduces the idea of residual network, which is to preserve the texture information of the detection object by shortcut connection. The advantage of this strategy is that it can enhance more useful information while suppressing irrelevant information.

This module can modify the kernel size and constructs groups network according to different instance objects. As a plug-and-play module, hybrid kernel module is suitable for multi-object detection and instance segmentation. The proposed method reduces the computational complexity of the FPN, resulting in a faster instance segmentation framework. The experiments show HKMask has better performance in the detection of low-resolution instance objects and good target extraction for complex background images. The proposed method achieves the best accuracy over the anchor-based method. We hope the simple and effective components will help the future instance segmentation task for low-resolution objects. Besides, some gradient-based projection algorithms [33, 34] can be also used to develop new methods for enhancing the image segmentation accuracy.

## Supporting information

S1 Fig The HKMask framework for instance segmentation. (TIF)

S2 Fig The schema of the original residual module (a) and the hybrid kernel module (b). Hybrid kernel module introduces attention mechanism and mixed convolution on the basis of the original Resnet module.
(TIF)

S3 Fig The improved squeeze-excitation networks. The max pooling is used to retain texture information and the average pooling retains global information of the feature map.
(TIF)

S4 Fig Original remote sensing image (a) and corresponding binary image (b).
(TIF)

S5 Fig Feature maps of three datasets in different stages. Compared to the original method, ours has a significant suppressing effect on unrelated background pixels, while enhancing the pixels of the target instance. This is conducive to the convergence speed in the process of model training.
(TIF)

S6 Fig Qualitative result of different methods on xBD and COCO datasets. (TIF)

S1 Table Details of ResNet-50 and hybrid kernel module-50. (PNG)

S2 Table The gains of ISE component in our design. Based on the framework of ResNet-50-FPN, results are reported on Balloon, COCO 2017val and xBD respectively.
(PNG)

S3 Table The gains of hybrid kernel component in our design. Based on the framework of ResNet-50-FPN, results are reported on Balloon, COCO 2017val and xBD respectively.
(PNG)

S4 Table Quantitative results on COCO 2017val. ConInst and BlendMask are implemented with Detectron2 and the object detection box AP (%) are reported.
(PNG)
