# Supplementary figures and images for: Instance segmentation convolutional neural network based on multi-scale attention mechanism
Source: PLoS One. 2022 Jan 27;17(1):e0263134. doi: 10.1371/journal.pone.0263134 (PMC8794127; doi:10.1371/journal.pone.0263134)

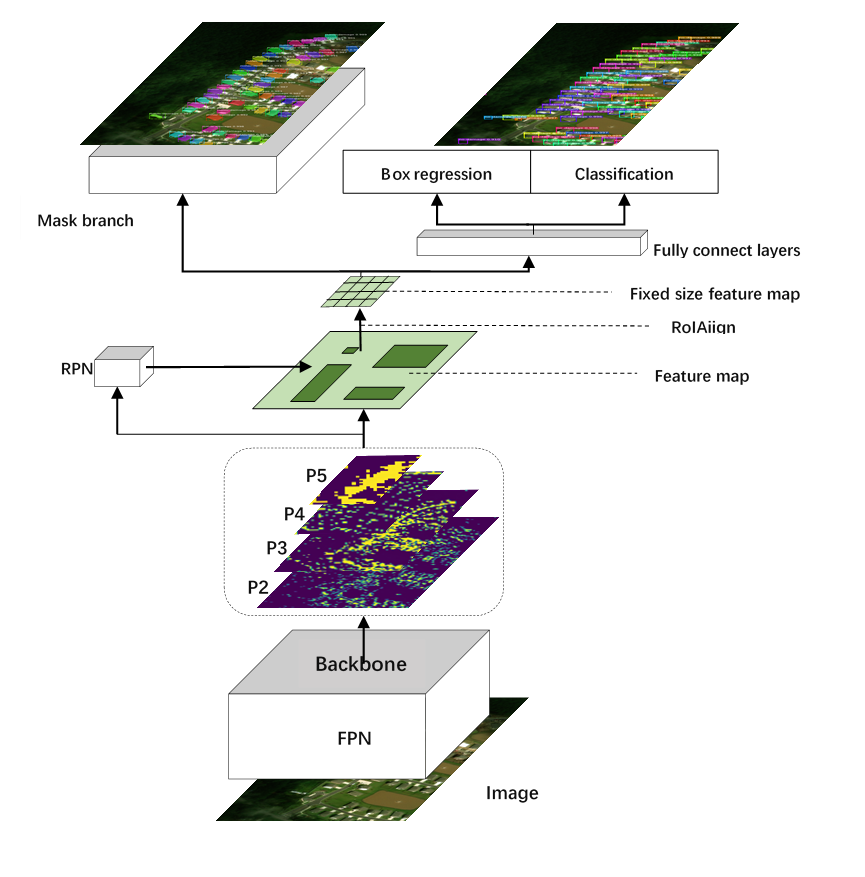

Supplement: S1 Fig — (TIF) [file pone.0263134.s001.tif]

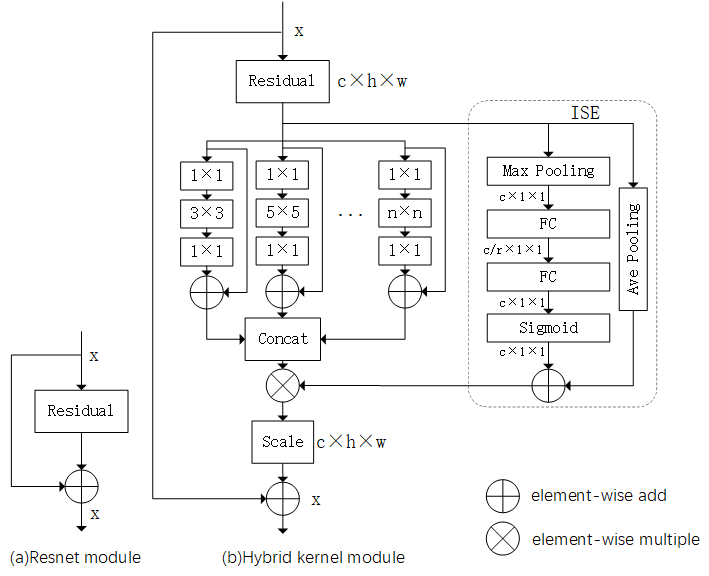

Supplement: S2 Fig — The schema of the original residual module (a) and the hybrid kernel module (b). Hybrid kernel module introduces attention mechanism and mixed convolution on the basis of the original Resnet module. (TIF) [file pone.0263134.s002.tif]

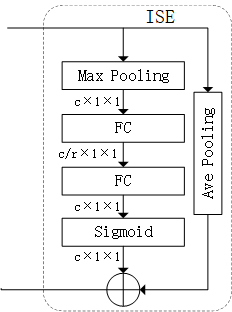

Supplement: S3 Fig — The max pooling is used to retain texture information and the average pooling retains global information of the feature map. (TIF) [file pone.0263134.s003.tif]

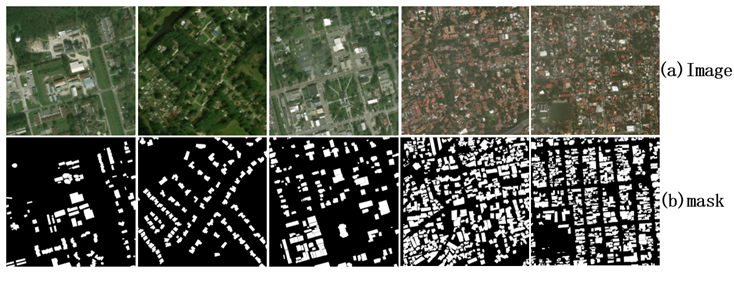

Supplement: S4 Fig — Original remote sensing image (a) and corresponding binary image (b). (TIF) [file pone.0263134.s004.tif]

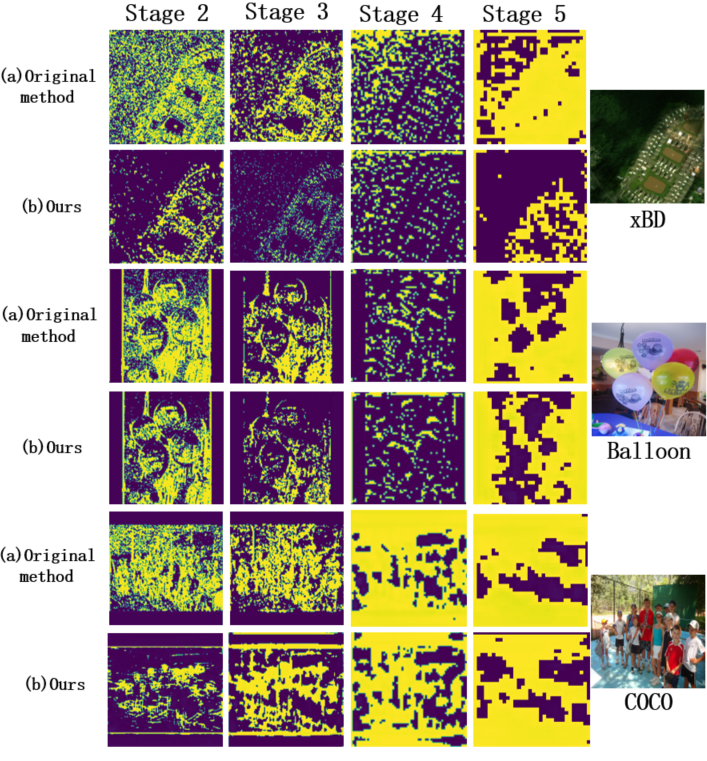

Supplement: S5 Fig — Compared to the original method, ours has a significant suppressing effect on unrelated background pixels, while enhancing the pixels of the target instance. This is conducive to the convergence speed in the process of model training. (TIF) [file pone.0263134.s005.tif]

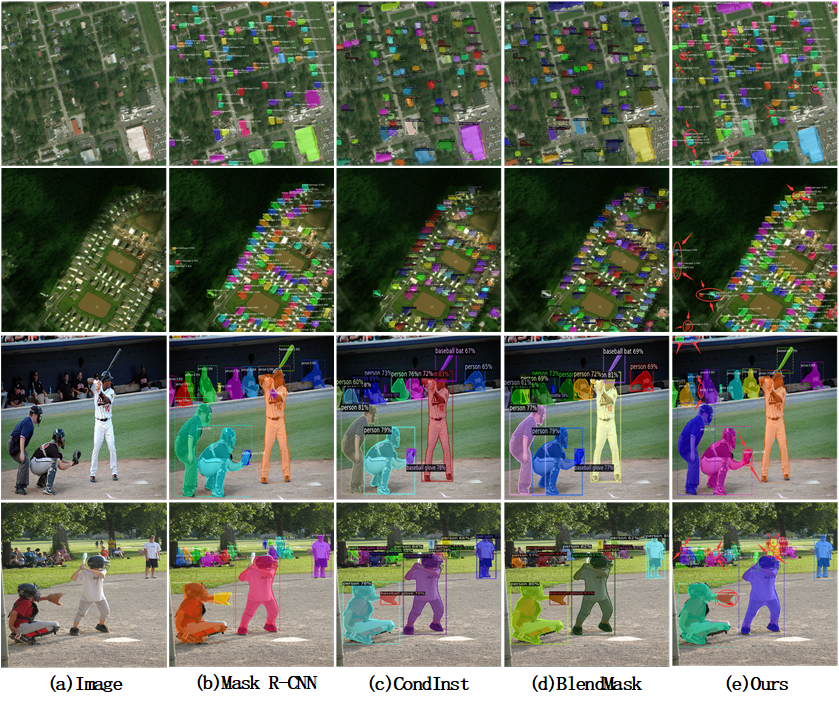

Supplement: S6 Fig — (TIF) [file pone.0263134.s006.tif]

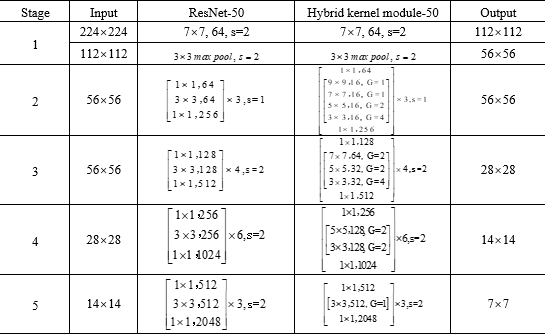

Supplement: S1 Table — (PNG) [file pone.0263134.s007.png]

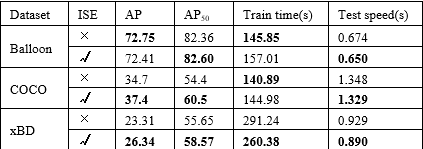

Supplement: S2 Table — Based on the framework of ResNet-50-FPN, results are reported on Balloon, COCO 2017val and xBD respectively. (PNG) [file pone.0263134.s008.png]

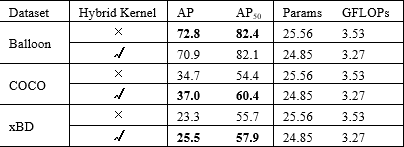

Supplement: S3 Table — Based on the framework of ResNet-50-FPN, results are reported on Balloon, COCO 2017val and xBD respectively. (PNG) [file pone.0263134.s009.png]

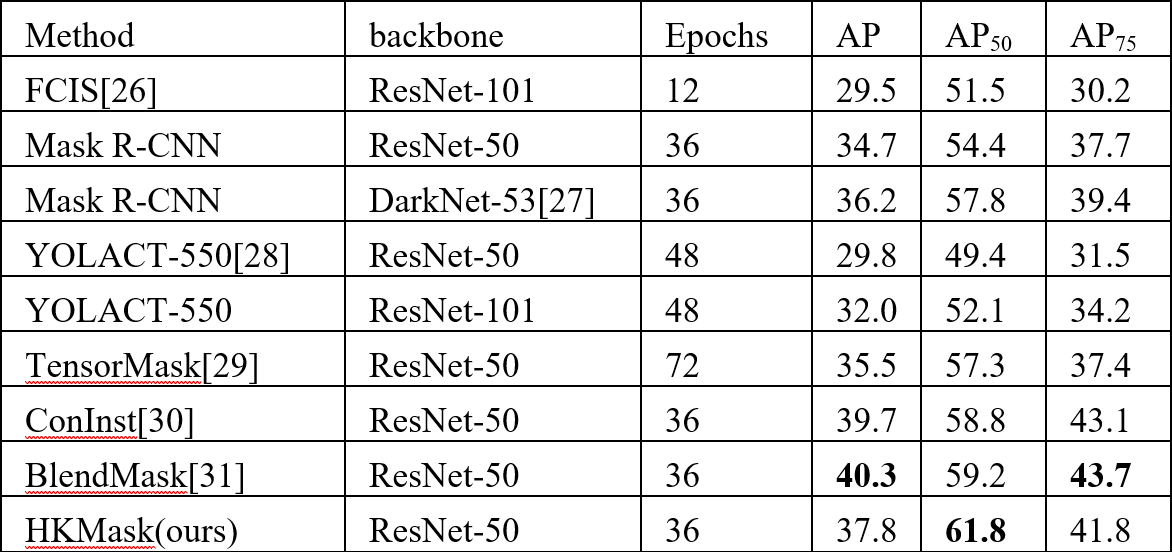

Supplement: S4 Table — ConInst and BlendMask are implemented with Detectron2 and the object detection box AP (%) are reported. (PNG) [file pone.0263134.s010.png]
